# Experimentally manipulated food availability affects offspring quality but not quantity in zebra finch meso-populations

**DOI:** 10.1007/s00442-022-05183-y

**Published:** 2022-05-25

**Authors:** Yoran H. Gerritsma, Merijn M. G. Driessen, Marianthi Tangili, Sietse F. de Boer, Simon Verhulst

**Affiliations:** University of Groningen, Groningen, Netherlands

**Keywords:** Brood size, Foraging, Growth, Life-history, Reproductive success

## Abstract

Food availability modulates survival, reproduction and thereby population size. In addition to direct effects, food availability has indirect effects through density of conspecifics and predators. We tested the prediction that food availability in isolation affects reproductive success by experimentally manipulating food availability continuously for 3 years in zebra finches (*Taeniopygia guttata*) housed in outdoor aviaries. To this end, we applied a technique that mimics natural variation in food availability: increasing the effort required per food reward without affecting diet. Lower food availability resulted in a slight delay of start of laying and fewer clutches per season, but did not affect clutch size or number of offspring reared per annum. However, increasing foraging costs substantially reduced offspring growth. Thus, food availability in isolation did not impact the quantity of offspring reared, at the expense of offspring quality. Growth declined strongly with brood size, and we interpret the lack of response with respect to offspring number as an adaptation to environments with low predictability, at the time of egg laying, of food availability during the period of peak food demand, typically weeks later. Manipulated natal brood size of the parents did not affect reproductive success. Individuals that were more successful reproducers were more likely to survive to the next breeding season, as frequently found in natural populations. We conclude that the causal mechanisms underlying associations between food availability and reproductive success in natural conditions may be more complex than usually assumed. Experiments in semi-natural meso-populations can contribute to further unravelling these mechanisms.

**Supplementary Information:**

The online version contains supplementary material available at 10.1007/s00442-022-05183-y.

## Introduction

Food availability is considered a key factor in life-histories and population dynamics, as survival and fecundity generally increase with food abundance (Martin 1987; Daan et al. 1989; Boutin 1990; Ruffino et al. 2014). However, direct effects of food availability on survival and reproduction are difficult to discern from indirect effects, as food availability also affects individuals through other ecological factors (McNamara and Houston 1987; Krebs et al. 1995; Prevedello et al. 2013). For example, food availability modulates local density of conspecifics, affecting disease dynamics (Adelman et al. 2015), predation risk through predator abundance (Gilroy and Sutherland 2007), and disease risk by avoiding food and foraging sites with a higher risk of exposure to parasites (Hutchings et al. 2001). Estimating direct effects of food availability on fitness components, i.e. independent of indirect ecological effects, is therefore challenging in a natural setting, but remains a necessary step to unravel the causal mechanisms underlying associations between food availability and phenomena on the individual and population level. The main experimental tool available to manipulate food availability in the wild is food supplementation, as experimentally reducing food availability is difficult to achieve. The effect of food supplementation on reproductive success has been extensively studied, and studies generally report increased reproductive success following food supplementation (Oro et al. 1999; Verboven et al. 2001; Davis et al. 2005; Peach et al. 2014), although others report no clear effects (Ewald and Rohwer 1982), or even reduced reproductive success (Nilsson 1994). A meta-analysis showed that food supplementation on average had a positive effect on reproductive parameters, but results were highly variable (Ruffino et al. 2014). However, interpreting food supplementation results has its complications, which may explain some of this variation. Firstly, unless food can be offered in a way that is not accessible to other individuals (e.g. inside a nest box, Verhulst 1994), the experiment may increase frequency of agonistic interactions as unintended side-effect. Secondly, unless the supplementary food is identical to the natural diet of the target species, the experiment is likely to change the quality of the food consumed in addition to the supplemented amount of food. This is likely, because the supplied food will usually be different from the natural diet, and because the supplied food will create room in the animal’s time and energy budget an individual can augment its diet with higher quality food compared to the control treatment, with potentially important effects on reproductive success (Tinbergen 1981; Williams 1996; Peach et al. 2014). Lastly, supplementary food is likely to change the optimal solution to the trade-off between selecting safe options with low rewards versus unsafe options with higher rewards (Godin and Smith 1988), leading food supplemented birds to experience lower risks of predation and infection, which may by itself benefit reproduction.

Thus, the mechanism through which food availability affects reproductive success is largely an open question; in particular to what extent this effect can be attributed to direct effects, as opposed to the array of potential indirect effects. Prior studies addressed this question by either manipulating the time (Lemon 1991; Wiersma and Verhulst 2005) or the effort required per unit food (Simons et al. 2014; Yap et al. 2021) and found reduced food availability to decrease reproductive success, mainly by decreasing laying intervals. In our study, we used a technique we developed to mimic natural variation in food availability, by manipulating the effort required per unit food, i.e. foraging costs (Koetsier and Verhulst 2011). We applied this technique year-round to populations of zebra finches (*Taeniopygia guttata)* housed in outdoor aviaries, provided with material for breeding, and studied foraging cost effects on reproductive success, i.e. number and growth of offspring. We refer to these populations as meso-populations, as in many respects they are between ‘populations’ of single captive individuals in laboratory environments and free-living populations. In this way, meso-populations form a bridge between biomedical and ecological studies. As group size was set by us, there was no confounding effect of density with increased food availability. Furthermore, predators were absent, there was no competition for food, and there was a single food source (diet) and hence little opportunity for variation in diet. We assumed therefore that any treatment effects are a direct consequence of variation in food availability.

The current experiment builds on an earlier experiment, in which we studied the effect of foraging costs on ageing and lifespan in non-breeding zebra finches (Briga et al. 2017). In that experiment, we tested whether growing up in harsh foraging conditions (many siblings) prepared birds better for coping with harsh foraging conditions in adulthood than growing up in benign foraging conditions (few siblings), as predicted by the predictive adaptive response hypothesis (Gluckman et al. 2005), or, alternatively, whether growing up in harsh foraging conditions resulted in birds of lower phenotypic quality, that were less able to cope with harsh foraging conditions in adulthood than birds grown up in benign foraging conditions, as predicted by the silver spoon hypothesis (Grafen 1988). Evidence on survival and lifespan clearly supported the silver spoon hypothesis, with individuals reared in benign conditions achieving a longer lifespan in ‘harsh’ foraging conditions in adulthood, while survival and lifespan were unaffected in benign foraging conditions in adulthood (Briga et al. 2017). In the current experiment, we created heterogeneity in phenotypic quality in the same way, by entering birds in the experiment that were reared in (manipulated) small and large broods in approximately equal numbers. We measured reproductive success in these birds for three consecutive years and here focus on female zebra finches to avoid the issue of extra-pair paternity.

We predicted that food availability would increase reproductive success, as individuals would be less constrained by the availability of food, allowing them to invest more energy in reproduction. We further predicted that birds reared in large broods, with a reduced life expectancy in harsh foraging conditions in adulthood (Briga et al. 2017), would increase their reproductive effort in response to the anticipated reduction in lifespan. This prediction is based on life-history theory stating that the optimal solution to the trade-off between reproductive effort and somatic maintenance will shift towards higher effort when residual reproductive value decreases (Kirkwood 1977; Kirkwood and Rose 1991; Boonekamp et al. 2020). Thus, we predict higher reproductive success for birds reared in harsh conditions (large broods), at least early in life, unless effort affects their state differently due to a different developmental background (McNamara et al. 2009), or when a higher effort is outweighed by a concomitant negative effect of developmental hardship on the efficiency with which reproductive effort affects reproductive success (e.g. an equal increase in reproductive effort does not translate into an equal increase in reproductive success). Such an effect would for example arise when birds reared in large broods are less efficient foragers. Furthermore, we predicted that reproductive success declines with age (Weladji et al. 2002; Adler et al. 2016; Hooper et al. 2017; Pei et al. 2020), due to the progressive loss of physiological and cellular function with age, and that this decline will be more prominent in individuals who were reared in harsh developmental conditions facing increased foraging costs during adulthood due to their reduced somatic state, as indicated by their reduced life expectancy, further accelerated by the anticipated treatment effect on reproductive effort.

## Materials and methods

### Birds

All breeding birds were reared in either ‘small’ or ‘large’ broods, as described in Briga et al. (2017). In brief, birds were reared by randomly paired parents housed indoors. All nestlings born were cross-fostered to either small (2–3 chicks) or large (5–7 chicks) broods, all within the natural range in the wild (Zann 1996), as well as in captivity (Griffith et al. 2017). Average brood sizes after cross-fostering were 2.6 (SD = 0.58, *n* = 48) and 5.5 (SD  = 0.59, *n* = 34) for small and large broods respectively. As described in detail in Driessen et al. (2021), growth rate from cross-fostering to day 15 was lower by 0.13 (range 0.10–0.17) grams per day in large broods; a notable difference as the average growth rate over all nestlings was 0.73 (SD 0.14) grams per day. Birds were kept with their parents until 35 days old, and subsequently moved to single-sex indoor aviaries (153 × 76 ×110 cm), together with four adults, two of each sex, to facilitate sexual imprinting. When approximately 100 days old, they were moved to the outdoor aviaries (320 × 150  × 210 cm).

Each of the four meso-population contained 18–24 adults, evenly divided over the sexes and developmental backgrounds (i.e. reared in small or large broods). Sample size was 67 females (35 benign; 28 harsh; four unmanipulated) and 66 males (29 benign; 34 harsh; three unmanipulated). At the start of the experiment, there was a wide age range, due to the time it took to build up the breeding population prior to the start of the experiment, but age at the start of the experiment did not differ between experimental categories. There was some mortality each year, and new birds were entered after each breeding season to keep the number of birds in each sex/treatment category approximately constant (Table 1). A small number of individuals entered in the aviaries to maintain numbers was reared in unmanipulated broods (usually because of absence of another brood for cross-fostering) and these were excluded from the statistical analyses.

**Table 1:** Number of individuals per treatment that were present at the start of each breeding season

Season	Developmental treatment	Foraging treatment				Total
		♀		♂		
		Benign	Harsh	Benign	Harsh	
2018	Benign	10	12	10	8	40
	Harsh	8	6	8	10	32
2019	–	0	4	0	3	7
	Benign	12	10	11	7	40
	Harsh	8	5	7	8	32
2020	–	0	3	0	3	6
	Benign	11	12	11	11	45
	Harsh	12	9	13	11	45

Note that seven individuals with no developmental treatment were added in 2019 (4♀ and 3 ♂). These individuals were not cross-fostered and therefore did not have a manipulated development. These birds were not included in the statistical analyses

### Foraging manipulation

We manipulated foraging costs as described in Koetsier and Verhulst (2011). In brief, a food box (120 × 10 × 60 cm) was suspended from the ceiling of the aviaries, with five holes on each side (resulting in 10 feeding positions). In the benign foraging treatment, perches were inserted beneath the holes, allowing the individuals to perch while eating. In the harsh foraging treatment, these perches were absent, forcing individuals to ‘hover’ and fly back to a distant perch to eat. Two aviaries had harsh foraging conditions and two aviaries had benign foraging conditions. Dropped seeds were inaccessible due to a trough under the food box. Prior to being entered in the experimental aviaries, all birds were trained for the foraging manipulation in a separate aviary, through daily shortening (1 cm total, 0.5 cm each side) of the perches until the individuals were not able to perch anymore. We previously showed that birds in harsh foraging conditions doubled the time spent foraging (Koetsier and Verhulst 2011).

For brevity, we refer to benign and harsh conditions with B and H respectively. For example, we refer to females that experienced benign conditions during development as BB females when they also experienced benign conditions in adulthood, and as BH females when they experienced harsh (foraging) conditions in adulthood.

### Reproduction

In each year, the breeding season was started in spring (2018: 9th of May, 2019: 15th of April, 2020: 20th of April) by installing 14 nest boxes (15 in 2018) in each aviary, together with nesting material (hay) in a wire mesh container on the aviary floor. Breeding seasons lasted until autumn, with the exact date depending on the termination of reproduction (date boxes removed: 2018; 7th of November, 2019; 1st of November, 2020; 23rd of October). Nest boxes were checked on Monday, Wednesday and Friday throughout the breeding season, to record nest building progress, number of eggs and/or nestlings, and measuring and ringing of nestlings. When no activity was recorded in a nest (no new eggs or hatchlings) for 21 days, all material was removed from the nest box. Clutch size per nest was determined as the number of eggs laid in a period of 21 days, with the first egg laid marking the start date. If new eggs were laid after this period, we recorded it as being a new clutch. Hatchlings were weighed when first observed to back calculate hatch date and hatchlings were marked by removing down feathers (Adam et al. 2014), to facilitate distinguishing individuals until they were ringed at the age of 12 days. At ages 15 (± 1) and 31 (± 1) days, we measured mass (to the nearest 0.1 g), wing length (to the nearest 0.5 mm), tarsus and head plus bill length (to the nearest 0.1 mm). At age 15 days, we also took a blood sample to establish genetic parentage (results will be reported elsewhere since we concentrate on female reproductive success here and female alternative reproductive strategies such as egg dumping occurred at very low rates of < 5 per year over all aviaries). After the measurement at age 31 days, individuals were moved from their natal aviary to another aviary where food was available *ad lib*, with four adults present, two of each sex. The offspring were moved at this age because parents reduced their feeding rate despite the offspring not yet being able to forage themselves from the food boxes.

All adults were uniquely color-ringed, allowing us to identify the social parents of each brood with visual observations. Out of the 459 chicks that reached the age of 15 days we were able to identify the social mother for 447 chicks (unknown identity for five chicks in 2018; seven in 2019; zero in 2020) and the social father for 435 chicks (unknown identity for 12 chicks in 2018; eight in 2019; four in 2020).

As clutches were frequently deserted before we could identify the social parents, we focused initial analyses on the reproductive output per aviary per season, divided by the number of social mothers present per aviary. Next, we concentrated the analysis of reproductive success on the number of offspring reared per social mother over the entire season and their growth. As we were interested in reproductive effort and the resulting success at rearing offspring rather than reproductive success on a genetic level, we took the number of offspring each female reared per breeding season as measure of reproductive success. We differentiated between the number of offspring reaching the age of 15 days (just before fledging) and the age of 31 days (removal from aviary). Interval from supplying the nest boxes to laying of a females’ first egg of the season (‘lay date’) was investigated as an additional aspect of reproduction, because time of breeding is associated with fitness in many populations (Verhulst and Nilsson 2008) and the focus of many food manipulation studies (Ruffino et al. 2014). However, because not all clutches could be assigned to a social mother, we compared the lay date of the first four clutches per aviary per year (48 in total) to test for food availability on the timing of the first clutch, as we could, with some certainty, assume that these clutches were produced by different parents.

### Statistical analysis

Analyses were done in R v 3.5.3 (R Development Core Team 2008) in the Rstudio IDE (v1.3.1073) (Rstudio Team 2020) using packages listed in Table S1.

To account for the age-variation among individuals when entered in the meso-populations, as well as variation in the subsequent exposure time to the foraging manipulation, we split the chronological age of an individual into two components: the age at which the individual was introduced to the meso-populations (“AgeStart”) and the time elapsed since that moment (“time in treatment”), which add up to chronological age. Individuals were introduced into the meso-populations on average at an age of 1.2 (SD 0.61) years.

Estimates of age effects within individuals are confounded by the progressive changes in population composition, due to selective (dis)appearance. Bias arising from this effect can be avoided by explicitly separating within-individual changes with age from the between-individual differences (selective disappearance; Van De Pol and Verhulst (2006) using within-subject centering, as shown by van de Pol and Wright (2009), with the function *gmc* from the *Rockchalk* package (v1.8.144, Johnson 2019). This function subtracts the subject’s mean value from each observation value (*X*_*ij*_* − *$\overline{X}$_*j*_), in our case the ‘time in treatment’ of an individual at the start of each breeding season subtracted by the mean time in treatment over all breeding seasons that it was active (minimum of one season, maximum of three). This variable (*X*_*ij*_* − *$\overline{X}$_*j*_) expresses the within-subject variation in age, where $\overline{X}$_*j*_ expresses the between-subject variation in age, where multiple observations of each individual (i.e. the breeding seasons) all have the same value $\overline{X}$_*j*_.

Eleven females died during the breeding seasons (2018; 4, 2019; 3, 2020; 4), which biases estimates when the dependent variable is reproductive success per breeding season. To avoid this bias, we included a covariate representing the proportion of the breeding season that the female was alive (1 = survived whole season, 0 = dead before the season started), scaled to the timing of offspring production in the aviary. For example, if there were 150 chicks within a season that reached the age of 15 days, and 90 individuals had been measured by the time a social mother died, her survival score would be 0.6.

### Analysis: lay date, clutch size and number of offspring

We used maximum log likelihood mixed effect models ((G)LMMs) with the lme4 (v1.1–21) and lmerTest (v3.1–0) packages (Bates et al. 2015; Kuznetsova et al. 2017) to analyse the data, with either a Gaussian or a Poisson distribution. We built separate models for the dependent variables: lay date, number of clutches, clutch size, number of hatchlings, and the number of offspring, separately for 15 and 31 days. In generalized LMMs, we further included an observation-level random effect (OLRE) to prevent overdispersion effects, following Harrison (2014).

For the analysis of annual reproductive output per aviary we divided the reproductive output per season by the number of social mothers present in that aviary, taken as the cumulative sum of their survival score. In this analysis, we included the foraging manipulation as fixed effect, and season (year) as random effect to account for the non-independence of repeated measurements of the same aviaries.

Next, we analyzed the reproductive output per social mother per season (*n* = 115 breeding seasons over 63 females). As fixed effects, we included the experimental manipulations, development and foraging conditions, as well as both the between-individual and within-individual changes in age. In addition, we tested the hypothesis that individuals with a shorter expected lifespan (i.e. individuals reared in large broods, and exposed to harsh foraging conditions in adulthood) increased their reproductive effort by including the variable “Expected Life Span” (ELS), which was coded one for HH females, and zero for all other treatment combinations (HB, BB, BH). As many of these females are still alive and reproducing, this coding relies on results we obtained earlier in the same setting (Briga et al 2017). We included the interactions between ELS and both age variables (within- and between-individual time in treatment) to test whether investment in lifespan changed with age when life expectancy is reduced. Additional non-experimental variables included were AgeStart and survival, and the random intercepts of the social mother and season to account for their non-independence. Initially, we included random intercepts of the aviary, but this explained little variance and was therefore excluded. All fixed effects were mean centered (mean = 0) which allowed for informative main effects despite their inclusion in interaction terms.

### Analysis: biometry

Because we measured multiple biometric traits that are strongly correlated, we used (Bayesian) multivariate response models fitted with the *brms* package (v2.14.4, Bürkner 2017), interfaced with the MCMC sampler of RStan (v2.19.2, Stan Development Team 2020). In addition to our focal variables, i.e. the experimental manipulations and time in treatment, we included age of the mother at entering the experiment, brood size and chick age (15 or 31 days). As we entered the aviaries thrice weekly, individuals were measured on the target age ± 1 day, and we therefore added the deviation in days from the target age as covariate to the models. We included interactions between foraging treatment and brood size and two-way interactions between ELS and both age variables. All other variables were entered as main effect only. The identity of the social mother and year of the breeding season were included as Gaussian random intercepts to account for their non-independence. Nest identity was initially included as random intercept, but explained a negligible amount of variance. We believe this is due to the inclusion of identity of the mother and the brood size, with these variables ‘capturing’ most of the variance associated with nest identity. For the measures of wing, tarsus and head-bill length, we included the identity of the person measuring the biometry as Gaussian random intercept to account for measuring bias.

All response variables were standardized (mean = 0, SD = 1) to facilitate comparison of effect sizes and to increase the efficiency of the MCMC sampler. The priors of all response variables were weakly-informative Gaussian priors (mean = 0, SD = 1) (Lemoine 2019). For the random effects, we employed the default priors of *brms*; Student’s *t* density with three degrees of freedom for standard deviations.

We ran the multivariate model on three chains with 1000 warmup iterations each, followed by 3333 sampling iterations, resulting in a total of 9999 iterations. Adapt_delta was set on 0.999 to prevent divergent transitions. Proper mixing of chains was checked with trace plots and convergence of chains by checking that Rhat values were (close to) 1.00. Model fits were evaluated by inspecting posterior predictive checks for each response variable, with the pp check() function in *brms.*

To test hypotheses, the probability of direction (*p*_d_) was calculated, defined as the proportion of the posterior distribution that is of the median’s sign, and represents the certainty that an effect goes into a particular direction (Makowski et al. 2019). The *p*_d_ is considered a Bayesian equivalent of the frequentist *p*-value (through the formula *p*_two-sided_ = 2 * (1 − *p*_d_) (Makowski et al. 2019).

Sample sizes for biometry are slightly lower than the number of offspring that reached the age of 15 or 31 days due to mortality on those days prior to the biometric measurements.

## Results

In total, 250 clutches resulted in one or more hatchlings (2018; 82, 2019; 81, 2020; 87) out of a total of 468 clutches (2018; 147, 2019; 165, 2020; 156). These 250 clutches yielded 573 hatchlings (2018; 184, 2019; 199, 2020; 190), of which 465 reached the age of 15 days (2018; 155, 2019; 164, 2020; 146), and 224 the age of 31 days (2018; 93, 2019; 68, 2020; 63) at which age they were removed from the experimental aviaries.

### Lay date, clutch size and brood size

Lower food availability delayed start of laying (*z* = 2.05, *p* = 0.04), with the first four clutches in the aviaries with benign foraging conditions laid 4.13 (SE 0.44) days after aviaries were supplied with nest boxes, compared to 5.42 (SE 0.50) days in aviaries with harsh foraging conditions.

Lower food availability significantly decreased the number of clutches per female per season (*t* = * − *3.21, *p* < 0.01, benign, mean ±  SE: 4.59 ± 0.26 clutches per female; harsh 3.43 ± 0.24). Females also laid more eggs per season in benign foraging conditions (*t* = −3.3, *p* < 0.01; benign, mean ± SE: 15.45 ± 1.24; harsh 10.61 ± 0.77), which was due to the larger number of clutches they started, as clutch size did not differ significantly between foraging conditions (*t* = −1.79, *p* = 0.11; benign, mean ± SE: 3.36 ± 0.15 eggs; harsh 3.09 ± 0.06). However, the number of hatchlings per female per season was not affected by the foraging condition (*t* = –1.32, *p* = 0.22; benign, mean ± SE: 5.94 ± 0.32 hatchlings; harsh 5.04 ± 0.60), which indicates that females were less successful in hatching their eggs in benign foraging conditions. However, while hatching success (i.e. proportion of eggs hatched) was indeed lower for benign foraging conditions, this did not reach statistical significance (*z* = 1.12, *p* = 0.26; mean ± SE: benign, 0.40 ± 0.04; harsh, 0.49 ± 0.07).

**Fig. 1:**
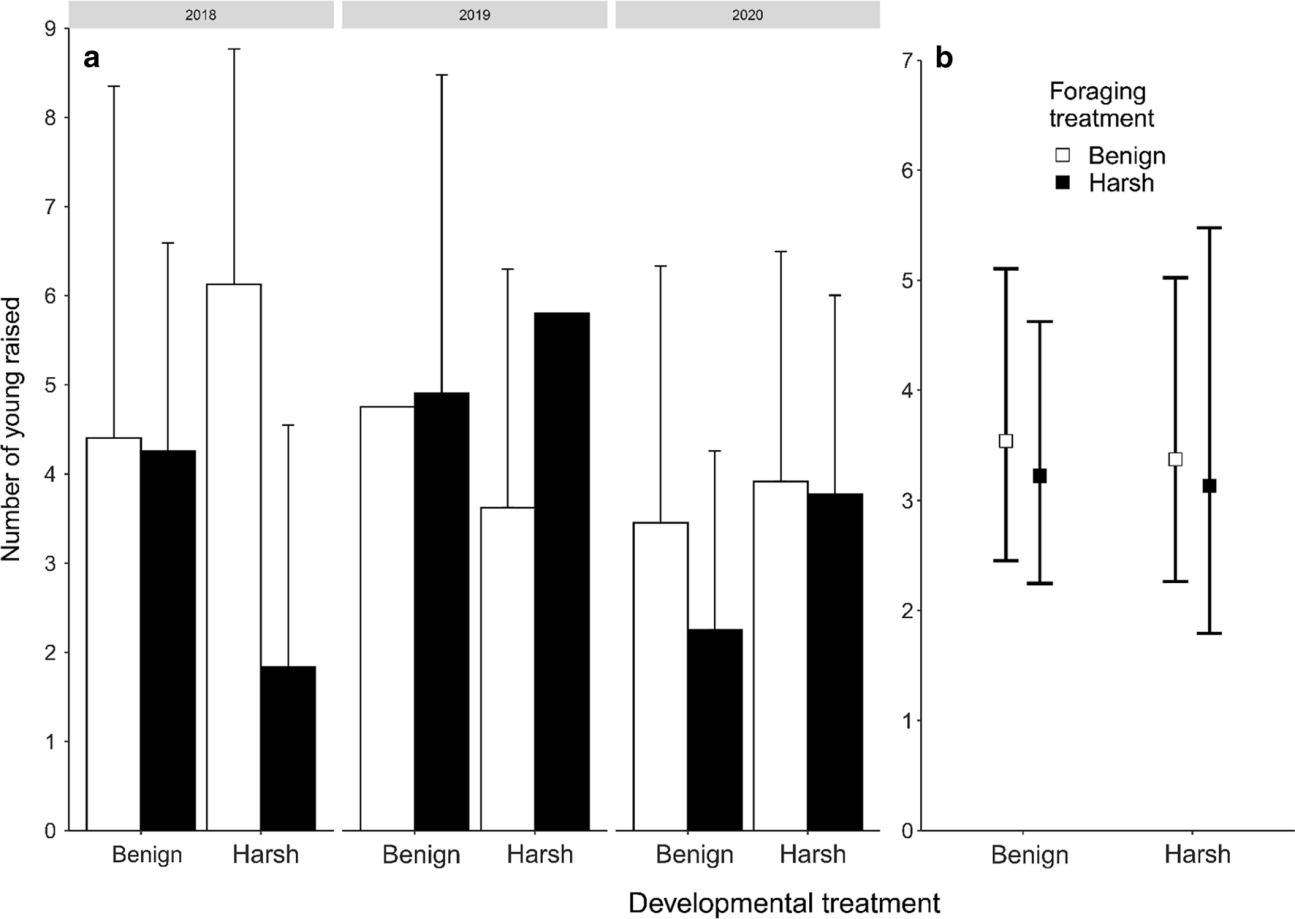
Offspring raised to the age of 15 days per social mother. **a** Number of offspring is given as the mean + SD, per social mother per season, **b** Number of offspring is given as the estimated marginal mean square and the 95% confidence interval per treatment group over all breeding seasons. Open bars/squares represent benign foraging conditions, whereas solid bars/squares represent harsh foraging conditions. Means and intervals are back-transformed from the log scale. Note that the axes are not identical

Next, we compared reproductive output per individual mother. Foraging costs had a non-significant effect on the number of eggs laid (*t* = −1.57, *p* = 0.12; benign, mean ± SE: 10.34 ± 0.85 eggs per female per season; harsh, 7.48 ± 0.67). Eggs were laid in 2.39 clutches per season on average, and this number did not differ between the foraging treatments (*z* =  −0.92, *p* = 0.36, benign, mean ± SE: 2.56 ± 0.20 nests; harsh, 2.20 ± 0.18). There was a trend for smaller clutches in the harsh foraging conditions (*t* =  −1.74, *p* = 0.09, benign, mean ± SE: 4.13 ± 0.20 eggs per clutch; harsh 3.46 ± 0.14) but this did not lead to fewer hatchlings (*t* = 0.08, *p* = 0.94, benign, mean ± SE: 2.18 ± 0.16 hatchlings per clutch; harsh, 2.23 ± 0.15). Foraging costs also had a non-significant effect on the number of hatchlings that reached the age of 15 days (Fig. 1, Table S4, *z* = −0.36, *p* = 0.72, benign, mean ± SE: 4.36 ± 0.43 hatchlings that reached day 15 per female per season; harsh, 3.72 ± 0.39) or 31 days (Fig. S1, Table S5, *z* =  −1.33, *p* = 0.18, benign, mean ± SE: 2.16 ± 0.30 hatchlings that reached day 31 per female per season; harsh, 1.70 ± 0.27).

Within-individual variation in age did not affect reproductive success (number of clutches: *z* = − 0.82, *p* = 0.41; eggs laid: *z* = −0.65, *p* = 0.52; number of hatchlings: z =−1.31, *p* = 0.19; number offspring raised to day 15: *z* = −0.80, *p* = 0.42, Fig S2). There was a negative trend for the offspring raised to day 31 (Fig. S3, *z* = −1.94, *p* = 0.05).

**Fig. 2:**
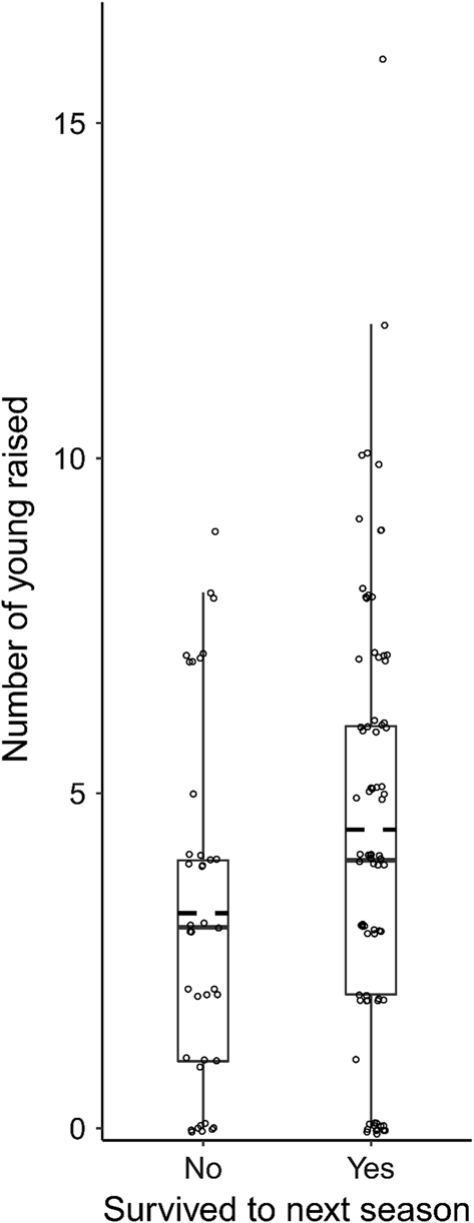
Females that survive better, raise more offspring. Each point denotes the number of offspring produced in one breeding season per social mother, respective to whether the mother survived to the start of the next season. The boxplots correspond to the interquartile range, where the solid line represents the median, and the dotted line the mean

In contrast to the within-individual age effect, between-individual variation in age was significantly associated with number of clutches (*z* = 2.5, *p* = 0.01), eggs laid per season (*z* = 3.6, *p* < 0.01), hatchlings (*z* = 2.24, *p* < 0.01), and the number of offspring raised to day 15 (*z* = 2.21, *p* = 0.03), with females with a higher average age having higher reproductive success. This association reflects that females that raised more offspring survived better to later breeding seasons. To illustrate this pattern directly, we applied a generalized linear mixed model with survival to the next season as a fixed factor, showing a significant association between survival and reproductive success (Fig. 2, *z* = 2.24, *p* = 0.03, surviving females, mean ± SE: 4.45 ± 0.37 offspring that reach day 15; non-surviving females: 3.21 ± 0.44). This effect weakened with offspring age, being no longer significant at the age of 31 days (*z* = 0.77, *p* = 0.44, surviving females, mean ± SE: 2.09 ± 0.28 offspring; non-surviving females: 1.66 ± 0.26 offspring). Effects of the between-subject variation in age did not differ between HH females and females from other treatments (clutches, *z* =  −1.23, *p* = 0.22; eggs laid, *z* = −0.41, *p* = 0.68; hatchlings, *z* = −0.18, *p* = 0.86; day 15 offspring, *z* = −0.02, *p* = 0.98; day 31 offspring, *z* = 0.63, *p* = 0.53).

### Offspring growth and survival

**Fig. 3:**
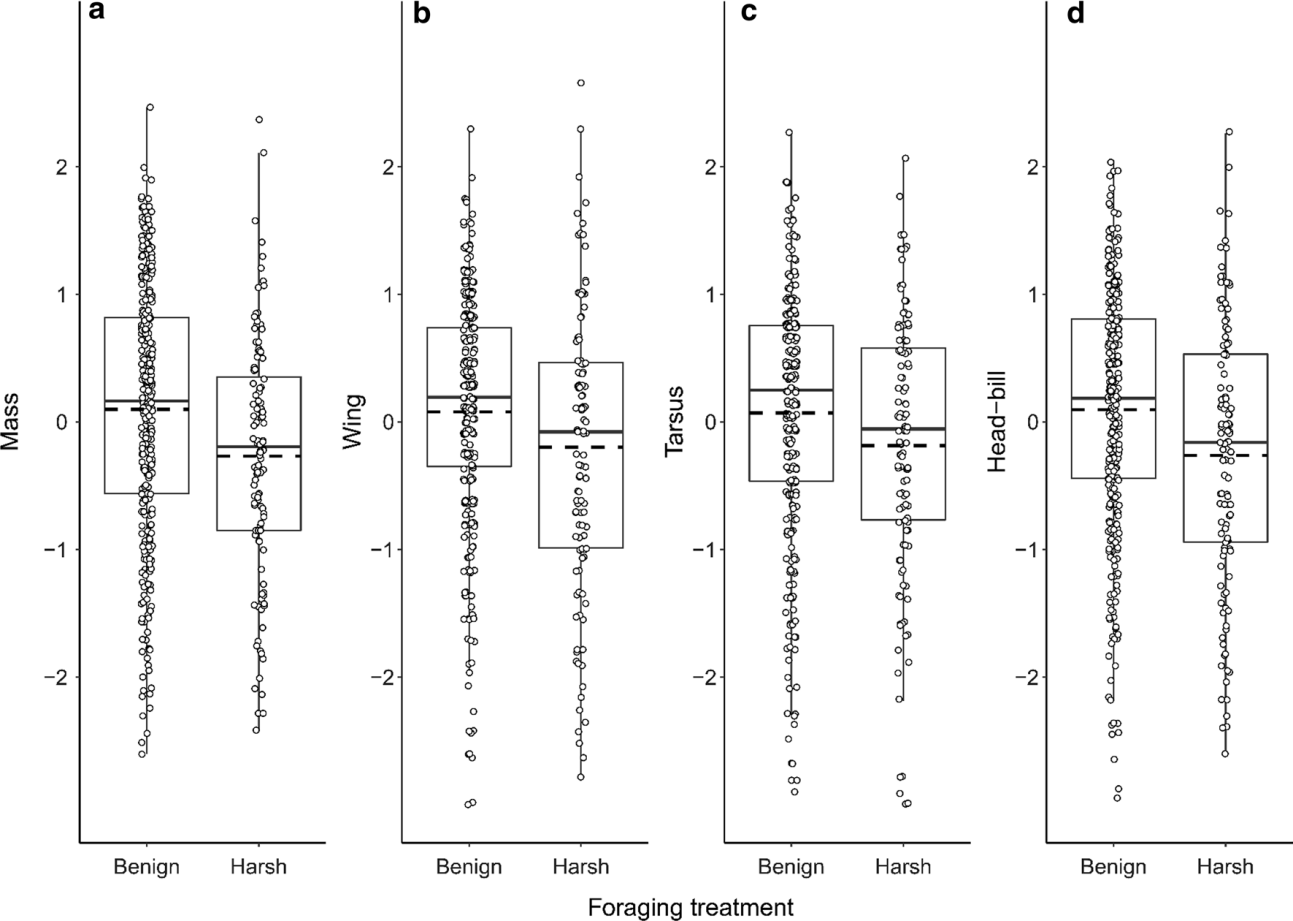
Foraging conditions negatively affects offspring growth at the age of 15 (± 1) days. Sample sizes for number of offspring are 328 in the benign, and 113 in the harsh foraging environment. Graphs show **a** mass, **b** wing length, **c** tarsus length, and **d** head-bill length. All measures are standardized (mean = 0, SD = 1). The boxplots correspond to the interquartile range, where the solid line represents the median, and the dotted line the mean

Growth was lower in harsh foraging conditions (Fig. 3). Specifically, at the age of 15 days, offspring growing up under harsh foraging conditions had lower mass, shorter wings and head-bill length (respective standardized effect sizes: −0.44, −0.39 and −0.45), and a trend for a negative effect on tarsus length (*p*_d_: 0.96; effect size:  −0.31). At the age of 31 days, offspring still had significantly lower mass (effect size:  −0.62), but effects on wing and head-bill length had weakened (effect sizes −0.26 and −0.04 respectively, Fig. S4). The age-dependent effect of foraging conditions on offspring growth could reflect compensatory growth of offspring in harsh foraging conditions, and/or biased mortality of smaller chicks in the harsh foraging conditions (these explanations are not mutually exclusive).

To test for enhanced compensatory growth in harsh foraging conditions, we compared the growth of wing, tarsus and head-bill length between day 15 and 31, where we included the foraging treatment and the time difference (in days) between the two measurements as fixed effects. Offspring reared under harsh foraging conditions grew their head-bill length significantly more than offspring from benign foraging conditions (*t* = 3.9, *p* < 0.01, benign, mean ± SE: 2.46 ± 0.07 mm; harsh, 2.90 ± 0.12) and there was a similar trend for wing length (*t* = 1.8, *p* = 0.07, benign, mean ± SE: 13.55 ± 0.32 mm; harsh, 14.43 ± 0.66). There was no effect of foraging conditions on compensatory growth of tarsus length (*t* =  −0.3, *p* = 0.72, benign, mean ± SE: 0.18 ± 0.04 mm; harsh, 0.14 ± 0.07), in line with the non-significant effect of foraging conditions on tarsus length at age 15 and 31 days.

**Fig. 4:**
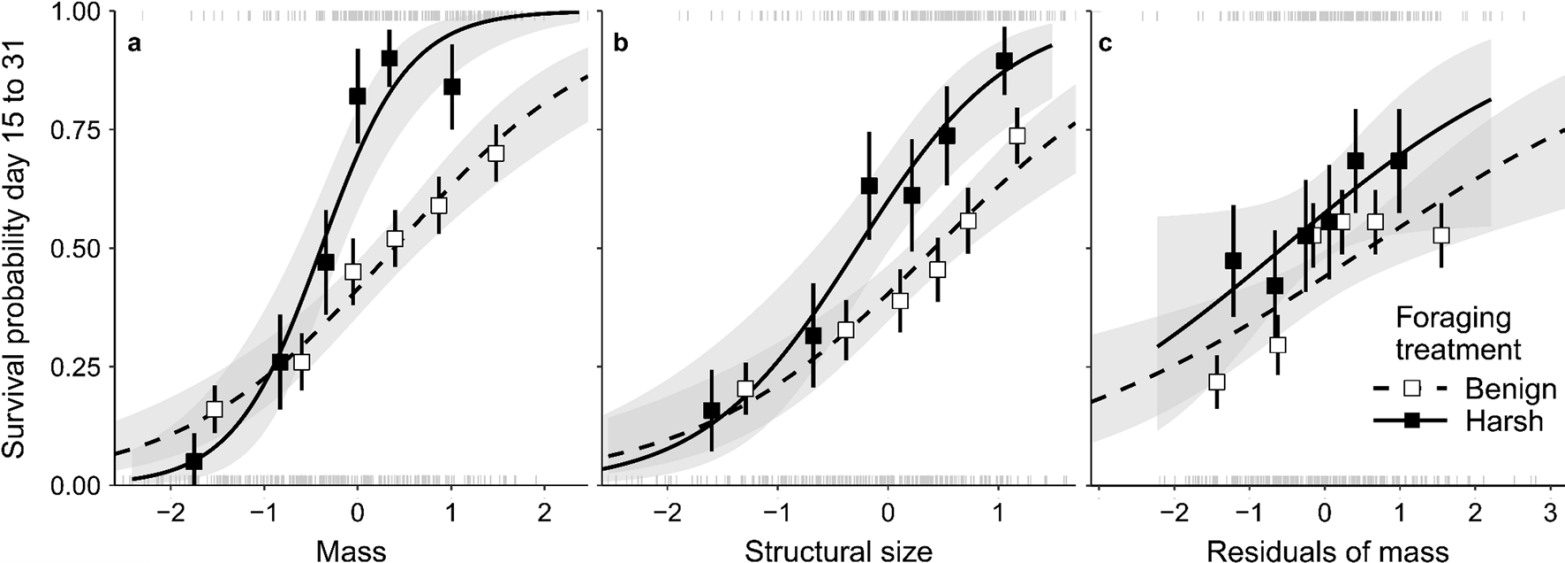
Survival depends on fledging mass and size, but not on residual mass. Each mark, at the top and bottom, represents an individual. Individuals are divided into six equal-sized categories, represented by boxes, based on their **a** standardized mass, **b** structural size, and **c** residuals of mass after correcting for structural size. Vertical whiskers represent the SE in survival probability. The black line is a Poisson smoother, with standard error in gray. Dashed lines and open boxes represent the benign foraging conditions, solid lines and filled boxes represent the harsh foraging conditions

To test for selective disappearance, we fitted a generalized linear mixed model with survival from day 15 to 31 as the (binomial) dependent variable, which revealed that higher mass on day 15 was associated with better survival to day 31 (*z* = 3.19, *p* < 0.01). However, this effect depended on the foraging treatment, with heavy chicks more likely to survive under harsh foraging conditions than under benign foraging conditions (Fig. 4A; interaction: *z* = 2.93, *p* < 0.01). This was due to chicks in harsh foraging conditions being smaller, because when correcting for structural size (using tarsus and head-bill, following Briga and Verhulst 2017) the foraging treatment effect was no longer significant (*t* = 0.29, p = 0.77), while the mass effect remained (Fig. 4B; *t* = 4.41, *p* < 0.01). Structural size also predicted survival (*z* = 7.5, *p* < 0.01), with larger offspring surviving better, and there was a trend for this effect to be stronger for offspring in harsh foraging conditions (interaction: *z* = 1.7, *p* = 0.09). Overall, survival was surprisingly low at this time, which we attribute to parents reducing provisioning rate while the offspring were not yet capable of self-feeding from the food boxes in either foraging environment.

**Fig. 5:**
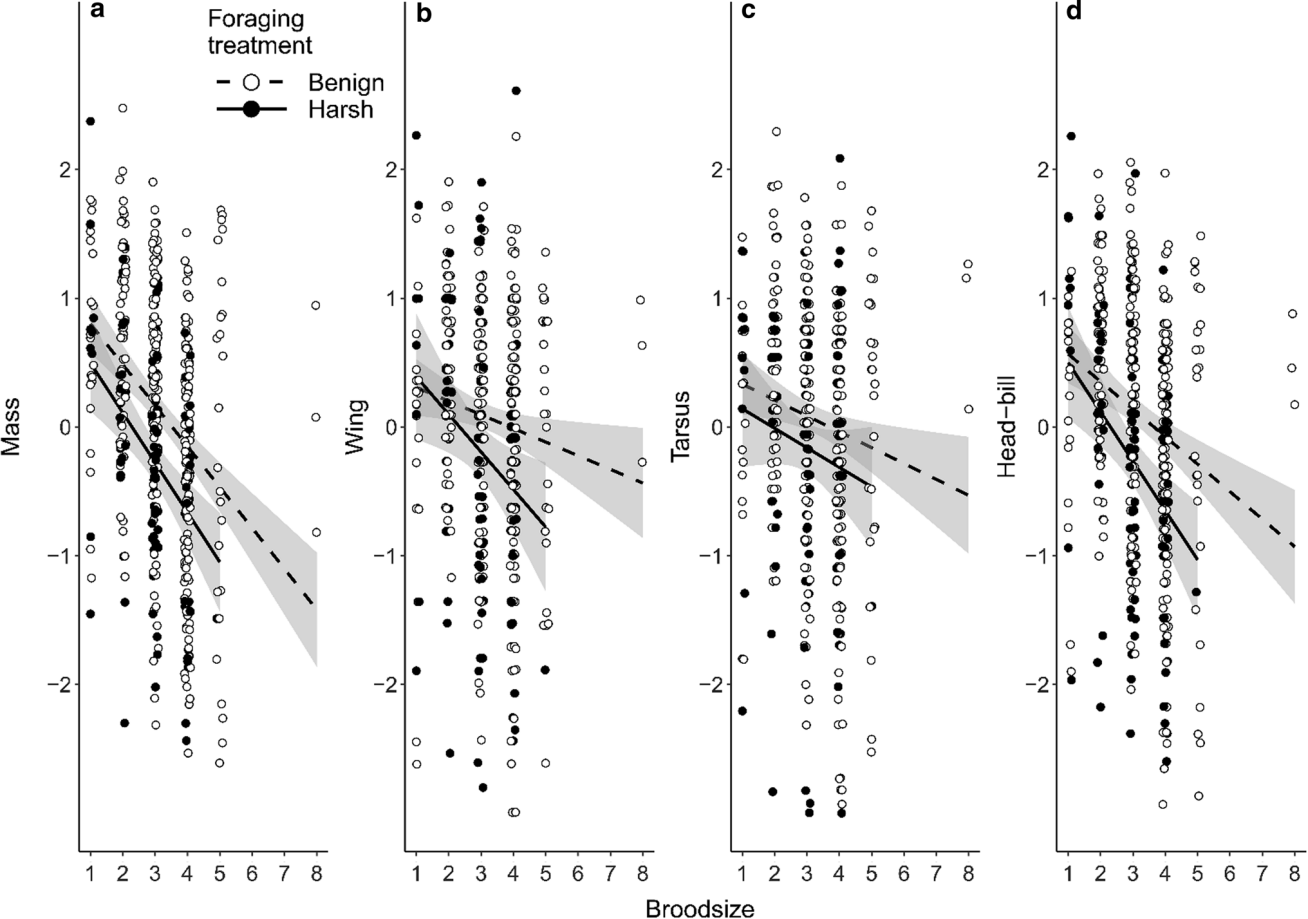
Growing up in larger broods negatively affects offspring growth, but does not depend on foraging conditions. Biometric values of the offspring at the age of 15 (± 1) days from the breeding seasons 2018–2020. Graphs show **a** mass, **b** wing length, **c** tarsus length and **d** head-bill length. All measures are standardized (mean = 0, SD = 1). Sample sizes, respectively for broods of 1, 2, 3, 4, 5, and 8 chicks, are 30, 85, 143, 152, 28 and 3. The black line is a linear smoother, with the SE in grey shading. The interaction between broodsize and foraging treatment was not significant for any biometric value

Independent of food availability, growing up in larger broods negatively affected all biometric measurements at the age of 15 days (Fig. 5, effect sizes of −0.39, −0.15, −0.18 and −0.31, respectively for mass, wing, tarsus and head-bill length), and all but wing length (effect size: −0.07) at the age of 31 days (Fig. S5, effect sizes of −0.16, −0.15 and −0.13, respectively mass, tarsus and head-bill length).

Maternal age (within-subject variation) did not affect offspring growth, with standardized effect sizes ranging from −0.22 to 0.11, all with CIs overlapping zero. Between-subject variation in age also was not associated with growth, with standardized effect sizes ranging from 0.04 to 0.21. The latter finding implies that there was no evidence for a difference in offspring growth between females that did or did not survive to the next breeding season.

### Females with shorter life expectancy

In contrast to our prediction, HH females showed no evidence of higher reproductive effort (Table S2). They did not lay larger clutches (*z* = 0.42, *p* = 0.68, HH, mean ± SE: 3.10 ± 0.26 eggs per clutch; other treatments 3.97 ± 0.14), or produce more hatchlings per season (*z* = 0.12, *p* = 0.91, HH, mean ± SE: 4.8 ± 0.73 hatchlings per season; other treatments 5.08 ± 0.39). Neither did the number of nests per season differ between HH and other females (z = 0.32, *p* = 0.75, HH, mean ± SE: 2.3 ± 0.29; other treatments, 2.4 ± 0.15). If anything, HH females raised fewer offspring to age 15 days than other females, yet without reaching statistical significance (Fig. 1; HH, mean ± SE: 3.7 ± 0.66; other treatments 4.1 ± 0.33; *z* =  −0.06, *p* = 0.95). They also did not raise more offspring to the age of 31 days (Fig. S1; *z* = 0.98, *p *= 0.33, HH, mean ± SE: 2.15 ± 0.51; other treatments 1.91 ± 0.23). The effect of ageing on reproduction was similarly absent in HH females as in females from other treatments (clutches, *z* = −0.292, *p* = 0.77; eggs laid, *z* = −0.71, *p* = 0.47; hatchlings, *z* = −0.75, *p* = 0.45; day 15 offspring, *z* = 0.03, *p* = 0.98; day 31 offspring, *z* = 0.54, *p* = 0.59).

Offspring growth did not significantly differ between HH females compared to offspring raised by females from the other manipulations, although effect sizes were in the expected (positive) direction, ranging from 0.26 to 0.51 at the age of 15 days, but with CI’s including zero (Table 2). At the age of 31 days, effect sizes ranged from −0.05 to 0.87, again with CI’s overlapping zero (Table S3).

**Table 2:**
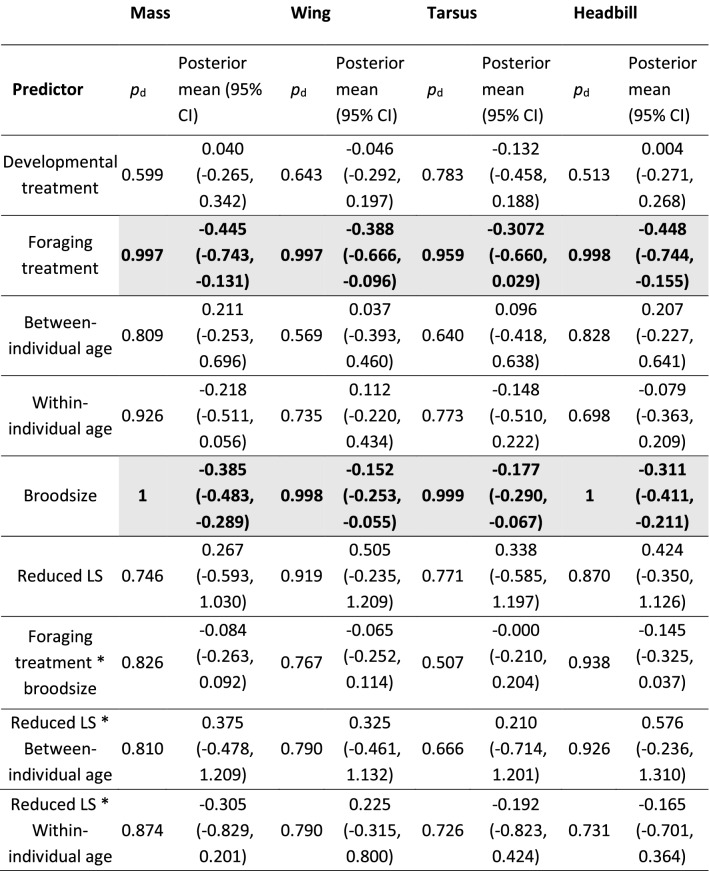
Marginal effects of all predictors and interactions on offspring size at the age of 15 days

Treatments correspond to the treatment of the social mother. For each posterior mean estimate, all other predictors were kept at their median (continuous predictors) or averaged over levels (factors). Variables not shown are deviation in age from 15 days and age of the mother when entering the experiment (“agestart”); both variables were also kept at their median value when calculating the posterior mean estimates. For broodsize and its interaction with foraging treatment, the effects are given per unit increase in broodsize. *P*_*d*_ values above 0.95 are gray-shaded and bold. Reduced LS (lifespan) indicates those mothers reared in large broods who subsequently bred in harsh foraging conditions

## Discussion

Food availability takes center stage in ecology, affecting organisms directly and indirectly in many ways. However, because of the confounds this generates, identifying direct effects of food availability under natural conditions is difficult. Manipulating food availability in the wild resolves this to some extent, but may in itself generate confounds. For example, it is generally difficult to provide extra food without also modifying diet, complicating interpretation of the experiment. We aimed to resolve these issues by manipulating food availability in what we label as ‘meso-populations’, using a technique that increases foraging costs without affecting the diet or other confounds. Our main finding was that lower food availability reduced offspring growth, without affecting the number of offspring reared. Reduced growth implies lower fitness prospects within the framework of our study, given that survival from age 15 to age 31 days depended on growth. Food availability also affected the start of laying, in agreement with a large body of food supplementation experiments in the wild (Ruffino et al. 2014) and prior studies in captive zebra finches (Wiersma and Verhulst 2005; Simons et al. 2014). The effect was small however, which we attribute to the constraint imposed by our experimental design, where the birds could only start reproduction after we supplied nest boxes.

Offspring growth declined with increasing brood size, and birds could therefore in principle mitigate the effect of the increased foraging costs on growth by raising smaller broods. It was unexpected therefore that brood size was independent of foraging conditions, given that optimality reasoning suggests that optimal brood size is smaller when foraging costs increase (e.g. Daan et al. 1990). On the other hand, the brood size effect on offspring growth was independent of food availability, suggesting that fitness of females in both treatments would benefit equally from an increase in brood size, explaining why brood size was independent of treatment. However, this argument implicitly assumes that the relationship between offspring growth and their fitness prospects is linear, which is unlikely, because the fitness benefits of an increase in size is likely to be larger for a nestling of intermediate size than for a large nestling. Indeed, we showed this to be the case for survival until nutritional independence (Fig. 4A), and propose that fitness of females subjected to harsh foraging conditions would have benefited from a smaller brood size. Apparently, females did not use food availability while laying as predictor of food availability during the nestling phase, which is in agreement with the observation that food supplementation in wild zebra finches did not stimulate reproduction (Zann et al. 1995). Instead, zebra finches have been suggested to base their reproductive decisions on other environmental cues such as rainfall (Zann et al. 1995) and ambient temperature (El-Wailly 1966; Ton et al. 2021), which may be better predictors of future breeding conditions.

Nestling mass and size at age 15 days were strongly affected by foraging conditions, but at age 31 days only mass was significantly lower in the harsh foraging environment; effects on size were smaller than at 15 days and no longer significant. The decrease in effect size with age was due to a combination of compensatory growth and selective disappearance of smaller chicks (although there was only a trend for the association between size and survival to differ between foraging conditions). While offspring in the harsh foraging environment were able to accelerate growth of wing and head-bill length relative to offspring in the benign foraging environment, thereby compensating for the growth-deficit accumulated at age 15 days, the effect of food availability on mass remained consistent until age 31 days (nutritional independence). Thus it appears that nestlings prioritized growth of size over growth of mass, which can be understood from a functional perspective, because size is fixed for life after the determinate growth period in zebra finches, whereas mass is a more flexible trait. Our finding of compensatory growth of morphological traits is in line with previous work in the zebra finch (Honarmand et al. 2010; Krause and Naguib 2014; Krause et al. 2017). Thus, while offspring size was not impaired by food availability at nutritional independence, they may still be negatively affected, as growth trajectories characterized by lower growth followed by compensatory growth are thought to come at a long-term cost (Metcalfe and Monaghan 2001), for example in their metabolic rate (Criscuolo et al. 2008), telomere attrition rates (Jennings et al. 1999) or lifespan (Birkhead et al. 1999). Thus it seems likely that offspring reared under harsh foraging conditions have lower fitness prospects at age 31 days, in part due to their lower mass, but additionally because of their growth-trajectory.

Zebra finches reared in an experimentally enlarged brood had a shorter lifespan when facing increased foraging costs in adulthood (Briga et al. 2017), raising the question whether reproductive output was also dependent on the interaction between treatments, as would be expected when both survival and reproduction reflect the same aspects of phenotypic quality. Following optimality theory, we predicted that individuals increase their reproductive effort when faced with reduced life expectancy (Parker and Smith 1990; McNamara and Houston 1996), a prediction supported by experimental studies (Bonneaud et al. 2004; Velando et al. 2006; Dawidowicz et al. 2010; Cotter et al. 2011; Bowers et al. 2012; Pietrzak et al. 2015; Sköld-Chiriac et al. 2019; Boonekamp et al. 2020). Previous work in zebra finches showed that individuals reared in enlarged broods delayed reproduction and produced less offspring but lived longer (Alonso-Alvarez et al. 2006). However, other studies in zebra finches reported that developmental conditions did not affect reproduction when the breeding environment was relatively benign (Tschirren et al. 2009; Krause and Naguib 2014). We anticipated that, as for survival, fitness differences would be larger in a harsh foraging environment, but instead find that there was no effect of developmental background on reproductive success regardless of foraging conditions. We note however that the absence of an effect of developmental conditions on reproductive success does not necessarily imply that there was no difference in reproductive effort, because multiple scenarios could give rise to this finding: one) individuals did not increase their reproductive effort following the experimental manipulation, i.e. a reduction in life expectancy, or two) individuals increased their reproductive effort, but their reproductive efficiency was lower due to their reduced phenotypic state, ultimately resulting in no change in reproductive success or three) individuals could not increase their reproductive effort, already having reached their ‘effort ceiling’ (e.g. Tinbergen and Verhulst 2000). A detailed study of reproductive behavior and reproductive effort is required to verify these scenarios, and to identify the extent to which constraint and restraint cause variation in reproductive success.

Reproductive success is often age-dependent, increasing with age in early life, likely due to increasing experience gained (Robertson and Rendell 2001; Rödel et al. 2009; Bouwhuis et al. 2015; Lv et al. 2016), and declining later in life due to physiological senescence (Forslund and Pärt 1995; Hayward et al. 2013; Nussey et al. 2013), and this decline may depend on (early) environmental conditions (Spagopoulou et al. 2020). Selective (dis)appearance with respect to reproductive success biases estimates of age-effects (van De Pol and Verhulst 2006), and we therefore mean centered age in our analysis. We found no evidence for reproductive success to vary with age in our study, but did find individuals with relatively high reproductive success to have better survival to the next breeding season. Higher mortality of poor breeders is also a frequent observation in natural populations (Bouwhuis et al. 2009; Nussey et al. 2011; Hayward et al. 2013; Kroeger et al. 2020; Brown et al. 2021). Such a pattern can arise through poor breeders living in habitats that are less suitable for both reproduction and survival, or because of low phenotypic quality of breeders affecting both reproduction and survival, or a combination of the two, with individuals of poor phenotypic quality ending up in poor quality habitats (e.g. Verhulst et al. 1997; van De Pol et al. 2006). However, in our meso-populations, we can rule out habitat differences as a cause of this variation, with all individuals having access to the same resources. Thus the higher mortality of poor breeders can be attributed to the phenotypic quality of the birds themselves. Apparently, there are phenotypic quality dimensions that are of importance for reproduction as well as survival, but from the current data set we cannot infer what these are. We previously showed diverse traits such as mass (Briga et al. 2019), glucocorticoid levels (Jimeno et al. 2018) and blood glucose levels (Montoya et al. 2018) to predict lifespan in the meso-populations, but whether these traits are also associated with reproductive success remains to be established. We note however that the meso-population setting we developed is ideally suited for this study, as it avoids the confounding effects of habitat quality that are unavoidable in natural populations.

## Supplementary Information

Below is the link to the electronic supplementary material.

Supplementary file1 (RAR 305 KB)

## Data Availability

Data are available on https://zenodo.org/record/5696990
